# How difficult is it to learn different steps of a cortical mastoidectomy?

**DOI:** 10.1007/s00405-025-09544-0

**Published:** 2025-07-08

**Authors:** Jesslyn Clarance Lamtara, Sudanthi Wijewickrema, Stephen O’Leary, Jean-Marc Gerard

**Affiliations:** 1 Department of Surgery (Otolaryngology), University of Melbourne, Melbourne, Victoria Australia; 2 The Royal Victorian Eye and Ear Hospital, Melbourne, Victoria Australia

**Keywords:** Cortical mastoidectomy, Temporal bone surgery, Virtual reality simulator, Difficulty level, Surgery training

## Abstract

**Introduction:**

A clear understanding of the difficulty levels of different aspects of a surgery is required to effectively guide and assess trainees. This study investigated which steps of cortical mastoidectomy are most challenging for novice and intermediate surgeons, and how these performances align with expert opinions.

**Methods:**

Thirty participants were divided into three groups: 10 novices (medical students), 10 intermediates (surgical trainees), and 10 experts (consulting ENT surgeons). Each performed cortical mastoidectomy on 8 anatomically different temporal bone specimens on a virtual reality platform. A senior otologist evaluated the performance using the validated Melbourne Mastoidectomy Scale (MMS). Additionally, 10 consulting otologists who are involved in training surgeons completed a questionnaire on the difficulty level of teaching the MMS items. Statistical comparisons between the novice, intermediate, and expert groups were conducted for each MMS item and compared with expert opinions.

**Results:**

Novices performed well only on defining the mastoid and entering the antrum. Intermediate group struggled mainly on sigmoid sinus related tasks and preserving the semicircular canals. Experts identified structural identification and defining MacEwan’s triangle as easier tasks, aligning with novice and intermediate performance. However, while experts perceived facial nerve related tasks and exposing middle fossa plate as the most difficult steps to teach, intermediate group performed comparably to experts on these.

**Conclusions:**

The contradictory results between intermediate group performance and expert opinion may result from training that emphasises complex mastoidectomy skills, at the cost of seemingly easier skills. These findings can be used for targeted feedback, assessment and curriculum development.

## Introduction

Cortical Mastoidectomy is a common otology procedure typically used to treat chronic otitis media with or without cholesteatoma. It is also the initial step of cochlear implant surgery and various lateral skull base operations. As such, it is a compulsory procedure in otology training [1, 2]. However, this is a challenging procedure, especially for trainees, due to the complex anatomy of the temporal bone and anatomical variability between individuals [3, 4]. During the procedure, the surgeon navigates around vital anatomical structures, such as the facial nerve, sigmoid sinus, dura of the middle fossa, semicircular canals, and incus [5]. Therefore, a good understanding of the three-dimensional (3D) positional relationship between anatomical structures [3], as well as high precision during the procedure is required, which imposes a significant cognitive load on surgeons [6]. As such, feedback and guidance during the surgery, as well as assessment of performance at its completion, are important factors when training this surgery. This will enable trainees to simultaneously grasp the concept of the standard being aimed for, compare their performance to the standard, and perform appropriate actions to lessen the gap [7].

In surgical training, feedback is usually provided verbally by experts in real-time and in the form of manually scored summative feedback at the end of the session. Surgical competency and technical skills are typically assessed using training assessment reports, oral examination and direct observation by expert surgeons [8]. As surgical education is moving towards competency-based assessment, the importance of accurate and effective assessment is increasing [9].

In recent years, with technologies such as virtual reality (VR) providing new surgical training platforms [10], there has been a move towards the assessment of temporal bone surgery in these environments. One approach has been to quantify drill movement. This relies upon analysis of the entire simulation and can differentiate between surgeons with different levels of experience [11–14]. The other approach has been to perform a summative assessment [9, 15–19], with the Welling Scale (WS1) being the most widely used [15]. These scoring scales typically assess the different aspects of the surgery using the final dissection [9, 15–17, 19] and/or task-based checklists of the dissection process [17, 18]. Other studies have explored the automation of validated assessment scales, including the Melbourne Mastoidectomy Scale (MMS) [19, 20] and a Modified Welling Scale [21].

In the provision of feedback or performance assessment, the identification of aspects of cortical mastoidectomy that are most difficult, is important; It will enable surgeons to focus on the more difficult aspects when providing guidance during training, more accurately weigh different aspects in assessment scales to reflect levels of expertise and develop more standardized training programs with differing levels of difficulty. Andersen et al. [22] and Nash et al. [23] identified the steps of a cortical mastoidectomy that novices found difficult. However, these studies were conducted on novice cohorts (medical students), with no performance comparison with intermediates (surgical trainees) or experts (consulting surgeons).

In this study, we address the gaps in the literature by systematically analysing the performance between three different skill levels, novice, intermediate, and expert, to identify the difficulty of each step in cortical mastoidectomy based on the MMS. By using multiple (8) anatomically different specimens in the study, we reduce the bias that may be present when using a single specimen. Furthermore, we compare these results with experts’ opinions of difficulty to determine how these correlates with novice and trainee performances. We anticipated poorer performance on steps of the mastoidectomy that trainer’s thought was more difficult to execute.

## Methods

### Study design

This is a prospective cohort study comparing the performance of novel, training-level and expert surgeons performing cortical mastoidectomy in a virtual reality surgical environment (The University of Melbourne temporal bone simulator). Surgical performance was assessed using the Melbourne Mastoidectomy Scale (MMS) [19]. The aims of the study were (a) to compare surgical performance on each item in the MMS across surgical expertise levels and (b) to compare these metrics with the perceived difficulty of each as judged by consultant surgeons active in surgical training. The hypotheses were:

1. Experts perform better than training-level surgeons, and trainees better than novice surgeons.
2. The performance of trainee and novice surgeons will be poorer on MMS scale items that experts judge more difficult to perform.

The study was conducted at the Royal Victorian Eye and Ear Hospital and approved by its Human Research Ethics Committee (HREC number 22/1527H). The design of this study was described in the previous study [11], and further analysis of the data was conducted.

As describe in Lamtara et al. [11], a total of 30 participants from 3 different surgical skill levels were recruited. The novice group consisted of 10 medical students from Victorian Universities with no prior surgical experience or AR/VR experience (educational or recreational). The intermediate group comprised 10 surgical registrars (residents) training in ear, nose and throat (ENT) surgery with knowledge and experience of the basic concepts of temporal bone dissection. Lastly, the expert group was 10 ENT consultants who were members of the Otology team at the Royal Victorian Eye & Ear Hospital. None of the participants had any prior exposure to any surgical training simulator. All participants performed a cortical mastoidectomy on 8 anatomically different temporal bone specimens. The temporal bone specimens used were all right sided, and the anatomically different specimens were numbered 1 to 8. For each participant, the order of the 8 bones was randomised to avoid scoring bias. The novice group was shown a video of how to perform a cortical mastoidectomy prior to drilling. No feedback was given to any of the participants during or after the dissections.

All procedures on the simulator were recorded with a screen capture software (Open Broadcaster Software, v0.569b). The end-products of the cortical mastoidectomy were anonymised and assessed by an ENT surgeon blinded to the group allocation using the MMS. This scale is an end-product assessment that uses a binary scoring system to evaluate a student’s performance on 20 different steps of the cortical mastoidectomy procedure (total score of 20).

In addition, to obtain expert opinion, 10 consultant surgeons specialised in Otology, all involved in surgical training, were recruited. They were asked to rate using a 3-point Likert scale (1-easy, 2-normal, 3-difficult) on the difficulty level of executing different steps of a cortical mastoidectomy, specifically when training registrars. The steps of the surgery were the 20 items in the MMS.

### VR simulator

The University of Melbourne temporal bone simulator was used in this study. Face and content validity of this simulator has been established [12, 24]. The virtual bones were generated using segmented micro-computerised tomography (micro-CT) scans of cadaveric temporal bones. The different segments of anatomical structures are rendered in different colours. A commercially available haptic device is used to mimic the behaviour of a surgical drill and provide force feedback (Geomagic Touch, formerly Sensable Phantom OMNI, 3D Systems, USA). Drill and virtual bone adjustments (such as burr size and magnification) are made through a MIDI controller.

### Statistical analysis

Each of the 20 MMS item scores was averaged across the 8 bones per participant. Then, these averages were compared between novice, intermediate and expert groups using the Kruskal-Wallis test on Matlab version R2023b (Mathworks, USA). P-values and effect sizes (Cliff’s delta) were obtained. Additionally, novice group performance scores for each MMS item were compared to existing study by Andersen et al. [22]. This was achieved by matching the items used in that to equivalent items of the MMS. Where multiple items used there related to a single MMS item score, the scores were averaged. Note that no statistical comparisons were possible due to differences in study design and scoring scales as well as limited data availability (from the previous study).

Inter-rater correlation coefficient (ICC) of the 10 expert raters was calculated using SPSS version 29 (IBM, Chicago, IL). The questionnaire responses were tabulated in Microsoft Excel and average scores and standard deviations for each MMS item were calculated. The relationship between mastoidectomy performance and expert opinion on the difficulty of the mastoidectomy steps were illustrated using linear mixed effect models on Matlab version R2023b (Matlabworks, USA).

## Results

### Performance comparisons between the 3 groups

**Fig. 1:**
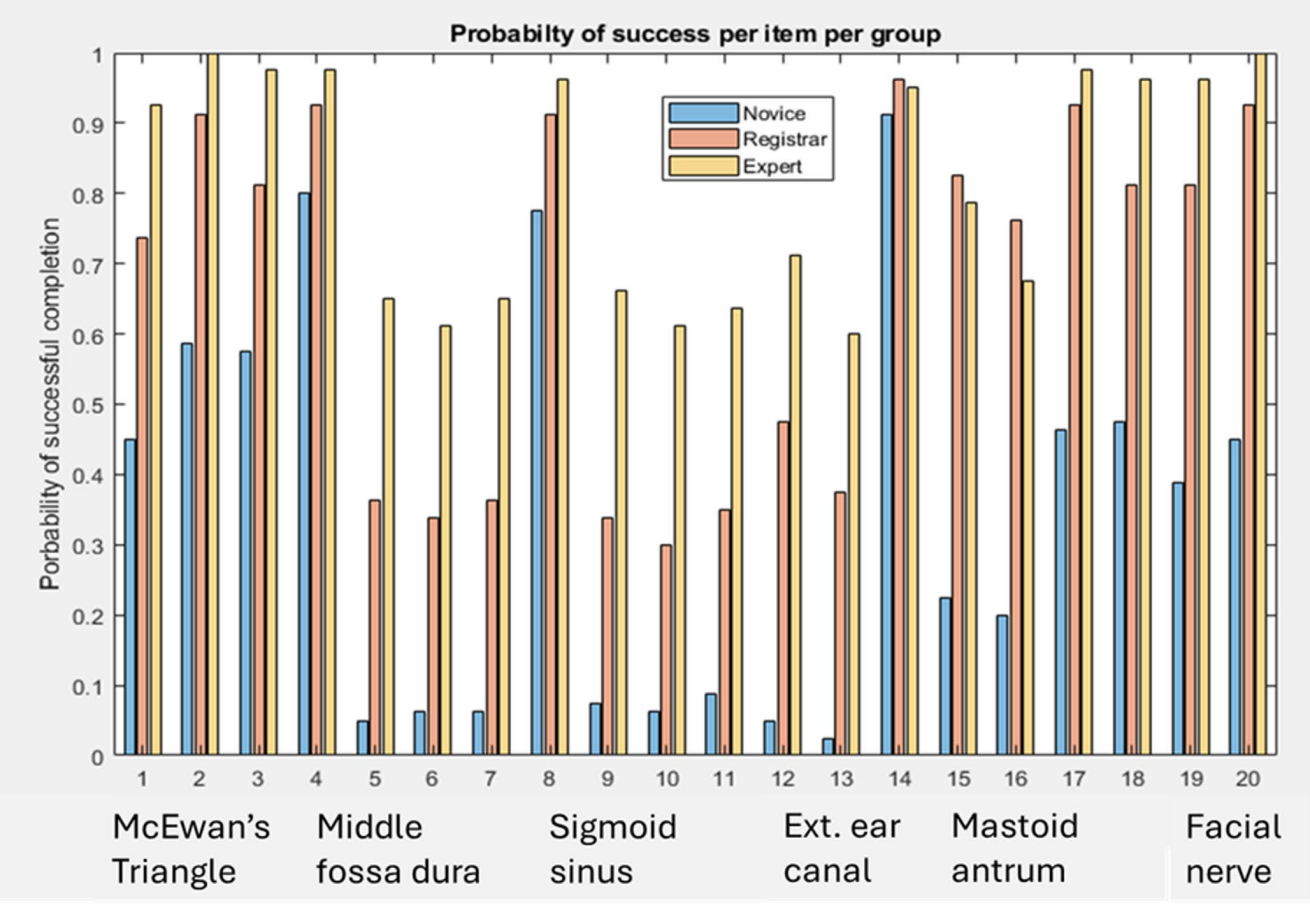
Performance for each Melbourne Mastoidectomy Scale (MMS) item for each group (novice, intermediate and expert groups) represented as the probability of successfully completing an item

When performances of all 3 groups were compared, it showed statistically significant differences (*p* < 0.001) for all items of the MMS, expect identification of middle fossa plate (MMS item 4) and preserving the external auditory canal (EAC) wall (MMS item 12). Figure 1 summarises performance on the MMS for each step of the cortical mastoidectomy. These steps are grouped by anatomical structure. The specific steps are summarised in Table 1. The performance of the novice group was consistently poorer than that of intermediate and expert groups at all stages of the mastoidectomy, except on identification of middle fossa place (MMS item 8) and preserving the EAC wall (MMS item 12). On the other hand, the intermediate group performed similarly to the expert group at most tasks. However, they struggled with sigmoid sinus related tasks (MMS items 3, 9–11) and preserving the semicircular canals (MMS item 16).

**Table 1:** *C*omparison of mastoidectomy performance between groups (novice, intermediate and expert) for each Melbourne Mastoidectomy Scale (MMS) items using the Kruskal-Wallis test. Statistically significant p-values are in bold text. Effect sizes of cliff’s delta are between brackets. All statistically significant results have large effect sizes.

		Novice vs. Intermediate		Novice vs. Expert		Intermediate vs. Expert	
		*p*-value (effect size)	Mean difference ± standard deviation	*p*-value (effect size)	Mean difference ± standard deviation	*p*-value (effect size)	Mean difference ± standard deviation
MacEwans Triangle defined as	1. Temporal line	**0.043 (0.53)**	0.288 ± 0.427	**< 0.001 (0.95)**	0.475 ± 0.293	0.179 (0.33)	0.188 ± 0.350
	2. Posterior external auditory canal wall	**0.015 (0.61)**	0.325 ± 0.450	**< 0.001 (0.8)**	0.413 ± 0.344	0.068 (0.3)	0.088 ± 0.167
	3. Sigmoid sinus	**0.042 (0.53)**	0.225 ± 0.436	**< 0.001 (0.98)**	0.413 ± 0.277	**0.04 (0.48)**	0.188 ± 0.238
Middle fossa plate	4. Identified	0.405 (0.2)	0.125 ± 0.368	0.056 (0.41)	0.175 ± 0.330	0.163 (0.28)	0.05 ± 0.134
	5. Adequately exposed^4^	**0.014 (0.61)**	0.288 ± 0.413	**< 0.001 (0.87)**	0.563 ± 0.369	0.079 (0.48)	0.275 ± 0.390
	6. Identified (adequately exposed) without minor damage^4^	**0.02 (0.56)**	0.275 ± 0.348	**< 0.001 (0.86)**	0.525 ± 0.362	0.078 (0.49)	0.25 ± 0.441
	7. Identified (adequately exposed) without major damage^4^	**0.02 (0.56)**	0.3 ± 0.392	**0.001 (0.87)**	0.563 ± 0.369	0.078 (0.48)	0.263 ± 0.435
Sigmoid sinus	8. Identified	**0.075 (0.45)**	0.138 ± 0.266	**0.016 (0.65)**	0.175 ± 0.179	0.728 (0.16)	0.038 ± 0.177
	9. Adequately exposed^8^	**0.009 (0.66)**	0.263 ± 0.341	**< 0.001 (0.98)**	0.575 ± 0.214	**0.018 (0.65)**	0.313 ± 0.278
	10. Identified without minor damage^8^	**0.006 (0.69)**	0.238 ± 0.246	**< 0.001 (0.97)**	0.513 ± 0.232	**0.011 (0.71)**	0.275 ± 0.293
	11. Sinodural angle defined^8^	**0.007 (0.68)**	0.263 ± 0.336	**< 0.001 (0.92)**	0.538 ± 0.250	**0.032 (0.57)**	0.275 ± 0.362
External auditory canal	12. Canal wall preserved	0.259 (0.26)	0.05 ± 0.087	0.265 (0.29)	0.038 ± 0.132	0.888 (0.07)	−0.0125 ± 0.092
	13. Posterior canal wall adequately thinned^12^	**< 0.001 (0.84)**	0.425 ± 0.383	**< 0.001 (1)**	0.638 ± 0.291	0.169 (0.4)	0.213 ± −0.339
	14. Canal wall thinned with no holes^13^	**< 0.001 (0.96)**	0.35 ± 0.211	**< 0.001 (1)**	0.563 ± 0.238	0.056 (0.54)	0.213 ± 0.257
Mastoid antrum	15. Antrum opened	**< 0.001 (0.93)**	0.426 ± 0.295	**< 0.001 (0.98)**	0.513 ± 0.285	0.148 (0.32)	0.05 ± 0.065
	16. Antrum opened with no damage of the semicircular canals^15^	**< 0.001 (0.94)**	0.475 ± 0.249	**< 0.001 (1)**	0.55 ± 0.197	**0.012 (0.5)**	0.075 ± 0.087
	17. Incus identified	**0.029 (0.57)**	0.338 ± 0.445	**< 0.001 (0.86)**	0.488 ± 0.325	0.065 (0.5)	0.15 ± 0.255
	18. Incus identified without damage^17^	**0.005 (0.73)**	0.425 ± 0.405	**< 0.001 (0.98)**	0.575 ± 0.265	0.065 (0.5)	0.15 ± 0.255
Facial nerve	19. Vertical section identified	**< 0.001 (0.88)**	0.6 ± 0.452	**0.002 (0.81)**	0.55 ± 0.501	0.603 (−0.05)*	−0.05 ± 0.222
	20. Identified with no damage^19^	**< 0.001 (0.89)**	0.55 ± 0.401	**0.005 (0.74)**	0.463 ± 0.483	0.486 (−0.12)*	−0.088 ± 0.221

Superscripted numbers (^1–20^) represent the dependency of that item on a previous item on the scale denoted by the number

*Negative value of effect size means negligible effect

The difference in performance between experience levels were quantified by the calculation of effect sizes (Table 1). All statistically different comparisons have large effect sizes of Cliff’s delta (> 0.47), except for sigmoid sinus identification (MMS item 8) which has a medium effect size.

Table 2 shows the mastoidectomy performance of the novice group for each MMS item and the study by Andersen et al. [22] which used a Modified Welling Scale. There is a similar overall trend in both studies. Where novices tend to perform poorly on similar items in both studies, suggesting that their skills may be independent of the specific VR environment. This applied particularly to items associated with damage to anatomical structures. For example, novices were observed to cause damage to the middle fossa and sigmoid sinus in both studies.

**Table 2:** Comparison of mastoidectomy performance of novices between this study and Andersen et.al [22] which used a Modified Welling Scale. Where the modified Welling scale used multiple items to address an MMS item, averages were used. All scores are out of 1

This study (2025) Melbourne Mastoidectomy Scale (MMS)			Andersen et al. (2017) [22] Modified Welling Scale		
		Novice performance (mean)	Novice performance (mean)		
MacEwans Triangle defined as	1. Temporal line	0.45	0.99	Temporal line	Mastoidectomy margins defined at
	2. Posterior external auditory canal wall	0.59	0.88	Posterior canal wall	
	3. Sigmoid sinus	0.56	0.98	Sigmoid sinus	
Middle fossa plate	4. Identified	0.8	0.98	Tegmen mastoideum exposed	Tegmen mastoideum/tympani
	5. Adequately exposed^4^	0.063	0.43	No cells remain	
	6. Identified (adequately exposed) without minor damage^4^	0.063	0.09	No holes	
	7. Identified (adequately exposed) without major damage^4^	0.063			
Sigmoid sinus	8. Identified	0.78	0.97	Exposed, no overhang	Sigmoid sinus
	9. Adequately exposed^8^	0.075	0.66	No cells remain	
	10. Identified without minor damage^8^	0.063	0.07	No holes	
	11. Sinodural angle defined^8^	0.088	0.56	Sharp, no cells remain	Sinodural angle
External auditory canal	12. Canal wall preserved	0.91	N/A	-	External auditory canal
	13. Posterior canal wall adequately thinned^12^	0.05	0.56	Thinning of the posterior canal wall, no cells remain	
	14. Canal wall thinned with no holes^13^	0.03	0.05	No holes	
Mastoid antrum	15. Antrum opened	0.46	0.99	Antrum entered	Antrum Mastoideum
	16. Antrum opened with no damage of the semicircular canals^15^	0.45	0.98	Lateral semicircular canal exposed and intact	
	17. Incus identified	0.48	N/A	-	
	18. Incus identified without damage^17^	0.39	N/A	-	
Facial nerve	19. Vertical section identified	0.23	0.94	Facial nerve identified (vertical part)	Facial nerve
	20. Identified with no damage^19^	0.20	0.14	No exposed nerve sheath	

Superscripted numbers (^1–20^) represent the dependency of that item on a previous item on the scale denoted by the number

### Expert opinion on the difficulty of each mastoidectomy step

Table 3 shows the responses from the 10 expert raters, rating the difficulty level of teaching cortical mastoidectomy steps based on the MMS. The ICC for the 10 raters was 0.898. Experts rated incus and facial nerve related tasks as the most difficult steps. In particular, identification of the vertical section of the facial nerve was rated as the most difficult task by the expert raters (average score = 2.8 out of 3), followed by adequate exposure of the middle fossa plate (average score = 2.6 out of 3). On the other hand, the expert raters thought that defining the temporal line of the MacEwan’s Triangle and identifying the sigmoid sinus (average score = 1.3 out of 3) were the easiest steps of a cortical mastoidectomy.

**Table 3:** Difficulty level questionnaire responses from the 10 expert raters. Ratings are: 1-easy, 2-average, 3-difficult. Items identified as difficult (an average score of over 2) are in bold

		Average scores (out of 3)	Standard deviation
MacEwans Triangle defined as	1. Temporal line	1.3	0.48
	2. Posterior external auditory canal wall	1.5	0.53
	3. Sigmoid sinus	1.5	0.53
Middle fossa plate	4. Identified	1.7	0.68
	5. Adequately exposed^4^	**2.6**	0.52
	6. Identified without minor damage^4^	**2.2**	0.42
	7. Identified without major damage^4^	1.8	0.63
Sigmoid sinus	8. Identified	1.3	0.48
	9. Adequately exposed^8^	**2.2**	0.63
	10. Identified without minor damage^8^	1.9	0.57
	11. Sinodural angle defined^8^	**2.4**	0.52
	12. Canal wall preserved	1.5	0.53
	13. Posterior canal wall adequately thinned^12^	**2.5**	0.53
	14. Canal wall thinned with no holes^13^	**2.4**	0.52
Mastoid antrum	15. Antrum opened	1.4	0.52
	16. Antrum opened with no damage of the semicircular canals^15^	1.6	0.52
	17. Incus identified	**2.4**	0.52
	18. Incus identified without damage^17^	**2.4**	0.70
Facial nerve	19. Vertical section identified	**2.8**	0.42
	20. Identified with no damage^19^	**2.5**	0.53

Superscripted numbers (^1-20^) represent the dependency of that item on a previous item on the scale denoted by the number

**Fig. 2:**
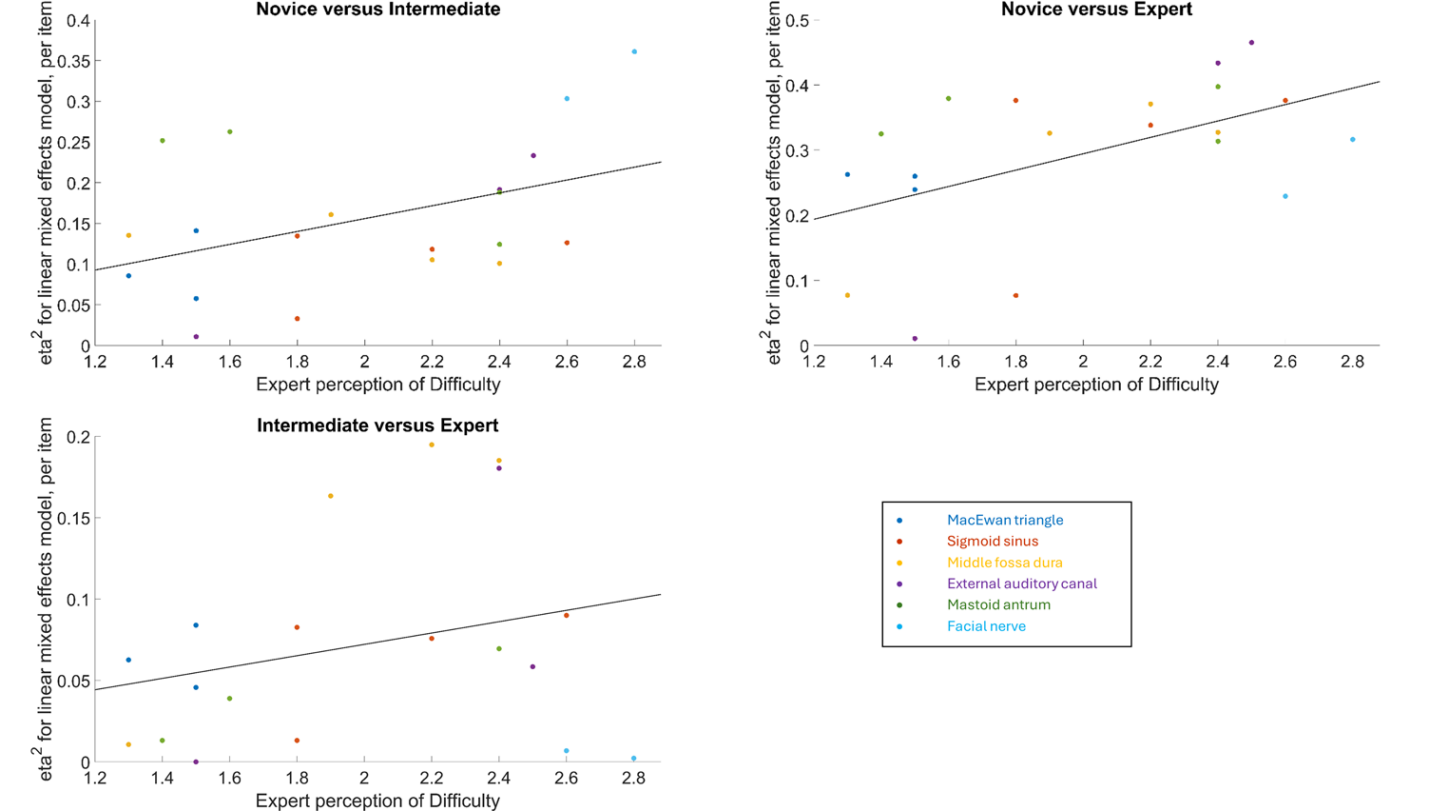
Relationship between the effect size (eta-squared) of groups (novice, intermediate and expert) mastoidectomy performance and expert perception of mastoidectomy steps difficulty, illustrated using linear mixed effects model. Group performances are represented for each Melbourne Mastoidectomy Scale (MMS) item and grouped by structures

According to Fig. 2 that illustrates the relationship between the effect sizes of novice, intermediate, expert groups mastoidectomy performance for each MMS item and expert opinion on mastoidectomy steps difficulty, there is a general trend for all comparisons: the more difficult a task is perceived to be by experts, the larger the effect size of the group’s comparisons.

## Discussion

### Dissection performance

The results of this study showed statistically significant differences for all MMS items, except for identifying the middle fossa plate (MMS item 4) and preserving the EAC wall (MMS item 12), when comparing the novice group to the intermediate and expert groups. These results were expected as the novice group lacks the surgical knowledge and technique to perform a cortical mastoidectomy successfully. They would have also found the transfer of knowledge from one specimen to another more challenging than the other 2 groups. The effect size of these differences was large. This reflects the gap in surgical knowledge and technical skills of novices, which is expected as novices have not yet received specialised training in temporal bone anatomy and surgical techniques.

Additionally, these results supported the findings of the study by Andersen et al. [22] where they concluded that generally, novices were unable to achieve high scores (scored using the Modified Welling Scale) when performing mastoidectomy on the Visible Ear Simulator. This study found that novices could easily complete tasks, such as defining mastoidectomy margins and entering the antrum. However, they struggled with more complex tasks, such as exposing the incus, adequately exposing the facial recess, avoiding damage to structures (sigmoid sinus, tegmen, and EAC), and leaving structures intact (lateral semicircular canal, ossicles, and facial nerve). Furthermore, in this study, we observed that mastoidectomy steps based on the MMS were recognised as difficult in general by the novices, and only middle fossa plate identification and EAC wall preservation was successfully completed. The differences in results between the two studies could be due to differences in study designs (e.g., provision of feedback during the procedures), skill levels of the cohorts, and the variety of specimens used.

In addition, similar results were also reported by Nash et al. [23], where they explored the performance of 4 novices (medical students) on learning to define mastoidectomy margins, and expose the sigmoid sinus and incus. They identified defining mastoidectomy margins and exposing the sigmoid sinus as easier tasks compared to exposing the incus. However, their analysis was not based on a validated assessment scale and was conducted on a single specimen using a novice cohort.

When intermediate and expert groups were compared, only sigmoid sinus related tasks (MMS items 3, 9–11) and not damaging the semicircular canals (MMS item 18) showed statistically significant differences. Additionally, these items had large effect sizes. This implies that the intermediates’ performance was still comparable to that of the experts, but further training is needed on sigmoid sinus-related tasks to reach expertise.

### Dissection performance vs. expert opinion

The inter-rater reliability was high (ICC = 0.898) indicating that the results in Table 3 are a good representation of experts’ views on how difficult different tasks are to learn in cortical mastoidectomy. These opinions can also be considered to be highly reliable as they were collected from consulting Otologists who are actively involved in training ENT registrars.

When looking at the relationship between group performance and expert opinion, there is a general trend where the more difficult a task is perceived to be by experts, the larger the performance difference between the trainee (novice and intermediate) groups and the expert group (Fig. 2). However, there were exceptions as detailed below. Expert opinion suggested that facial nerve related tasks (MMS item 19–20) and adequately exposing middle fossa plate (MMS item 5) were perceived by experts to be the most difficult steps to teach in cortical mastoidectomy. However, intermediate group performance on these items was comparable to expert group performance in the VR environment. This could be because of trainers’ continuous emphasis on facial nerve and middle fossa tasks during training. Interestingly, the intermediate group struggled with sigmoid sinus related tasks, which were thought to be easy-medium tasks by the experts. This shows that trainees might need more focused training on sigmoid sinus tasks. These types of observations show the strength of breaking down the tasks to look where the emphasis should be while teaching.

Experts perceived most structure identification tasks and defining MacEwan’s triangle as easier tasks. This is consistent with the comparison results, as intermediates performed on par with experts on these tasks. This suggests that trainers are employing a robust teaching method for these cortical mastoidectomy steps.

### Implications for surgical training

The results of this study may have practical implications in surgical training in the VR environment, and possibly more broadly. By comparing expert opinion with trainee performance, we gained insight into current training practices and their outcomes. For example, some tasks that experts considered to be easy, and therefore, presumably did not focus on when training registrars, were found to be difficult for them to complete well. Similarly, there were a few tasks that experts considered important and difficult, which registrars were able to complete well, likely due to the constant emphasis and focus during training. As such, the results will lead to a shift in how difficulty levels of surgical steps are viewed and change how feedback is provided during training.

When developing competency-based assessment criteria, it is valuable to understand which steps of a procedure are more difficult in order to weigh them appropriately. These results are equally applicable in shaping the nature of expert feedback and assessment during traditional surgical training as well that of automated feedback and assessment in simulated training environments. Furthermore, the results will assist in building specific training tasks, such as repetition of certain steps (e.g. temporal line). This will lead to the development of more effective and efficient surgical curricula.

### Limitations

One of the limitations of this study is that the number of participants was relatively small (10 participants per group with a total of 30). A larger group sizes would provide more detailed and precise results. However, given that the effects seen were large (0.7-1; Table 1), it is likely that the study had sufficient power to provide confidence in the validity of the outcomes, as estimated from G*Power version 3.1.9.7 (F-test, effect size 0.8, α = 0.05, total sample size 30, number of groups = 3).

The effect of learning through repetition was not considered, whereas literature suggests that repetition without feedback may still be effective in increasing some aspects of cortical mastoidectomy performance in registrars [14]. However, the results of this previous study were not based on a validated assessment scale and therefore, are not directly comparable. Furthermore, most of the intermediate group’s results were significantly different to that of experts, suggesting that the learning effect of repetition was negligible after 8 repetitions. This is consistent with the results of a previous study which showed that trainee (novice and intermediate) performance did not reach expert levels after 8 repetitions [11, 25]. One reason for the failure to reach expert levels after 8 repetitions could be due to the lack of feedback, which has been shown to be an important aspect of skill acquisition and retention [26].

Although the 8 specimens used in this study were all healthy and normal, their anatomical variation may have caused some aspects of the surgery to be more difficult to perform on some specimens when compared to others. Additionally, cortical mastoidectomy steps were based on the MMS. Although it has been validated and recognised in literature, there might be other factors (other medical conditions or environment factors) that could influence the difficulty level of a cortical mastoidectomy. However, the identification of the complexity of surgical steps in general, regardless of the specifics introduced by anatomical variation is still valuable, as this mimics real-life training settings where cadaveric specimens are used in training.

Moreover, the use of VR platform could also influence the sensitivity of the comparisons. Even though the VR simulator used in the study has been validated and proven to significantly improve mastoidectomy performance, there are still some differences compared to real-life experience, such as fidelity and visual cues [27]. Therefore, these may limit the generality of the results.

In this study, for the sake of analysis, difficulty of an item was associated with how well it was completed. However, in practice, the situation may be more complicated. For example, an item that is perceived as difficult may have led to more focused training of that item resulting in trainees gaining competence in it faster.

## Conclusion

Feedback and assessment are crucial aspects of surgical training. To provide effective feedback and assessment, as well as to develop surgical curricula, it is important to identify which components of the training trainees find difficult. This study differentiated difficulty levels of mastoidectomy training steps based on the MMS by comparing the performance of novice and intermediate groups to that of expert groups. Although there were some correlations between expert opinion and study results, there were important differences as well, challenging some long-standing experts’ perceptions on otology training. For example, focusing on some aspects of a surgery that are seen as riskier (e.g. facial nerve), without paying enough attention to teaching basic principles (e.g. defining MacEwan’s triangle) may be detrimental. Further studies to look at whether similar patterns appear on other surgical learning media, such as cadavers and additive manufactured temporal bones, would also be beneficial to further improve/confirm current surgical curricula.
